# Successful Smoking Cessation and Duration of Abstinence—An Analysis of Socioeconomic Determinants

**DOI:** 10.3390/ijerph7072789

**Published:** 2010-06-30

**Authors:** Joachim Marti

**Affiliations:** Institute for Research in Economics, Economics Department, University of Neuchâtel, Avenue du 1er Mars 26, 2000 Neuchâtel, Switzerland; E-Mail: joachim.marti@unine.ch; Tel.: +41-32-718-1406; Fax: +41-32-718-1401

**Keywords:** socioeconomic status, inequalities, smoking cessation, abstinence, duration analysis

## Abstract

Smoking does not affect every socioeconomic subgroup of the population equally, resulting in major inequalities in terms of smoking-related morbidity and mortality. While previous studies mainly focused on inequalities in smoking prevalence, we have analysed the socioeconomic dimensions that might be associated with two other smoking-related outcomes: the odds of successfully quitting and the duration of abstinence. Using nationally representative Swiss data, we found evidence of a socioeconomic gradient in successful cessation and abstinence duration with respect to education level and income for both men and women.

## 1. Introduction

In Switzerland, we have observed a sharp decline in smoking prevalence from 33% in 1997 to 28% in 2007 (these prevalence rates refer to the proportion of smokers (regular and occasional) in the Swiss population aged 15 and over). Over the same time period, we have also noticed a 7% increase in tobacco-related mortality among women, while men experienced a 10% decrease in tobacco-related mortality. Similar figures have been reported in most developed countries and are consistent with the tobacco epidemic model proposed by Lopez *et al.* [1], in which the authors break down the nationwide diffusion of tobacco use into four distinct stages. The last stage is characterised by a decrease in the prevalence of tobacco use for both men and women but an increase in tobacco-related mortality among women due to the lag between tobacco use and tobacco-related mortality. Another important feature of the diffusion of the epidemic is the widening of socioeconomic differences in the context of smoking prevalence. In the early stages of the process, smoking prevalence was higher in upper socioeconomic groups. Today, however, this trend has reversed, resulting in major socioeconomic inequalities in terms of both smoking prevalence and smoking-related morbidity and mortality.

Smoking has been identified as a primary cause of inequalities in death rates between different social classes [2]. In a study conducted among European men, Mackenbach *et al.* [3] found that 20% of the educational differences in those who suffered premature mortality were attributable to smoking. Extensive international literature offers evidence that tobacco does not affect all socioeconomic subgroups of the population equally (it is estimated that smoking prevalence is about 50% higher in lower socioeconomic groups than in higher groups [4]). Giskes *et al.* [5] analysed trends in smoking behaviour by education level between 1985 and 2000 in Western Europe. They found a greater decline in smoking prevalence and consumption levels among more educated individuals. Huisman *et al.* [6] also found that education was a strong predictor of smoking in Europe. In a study among British women, Harman *et al.* [7] identified socioeconomic gradients for ever-smoking, quitting and current smoking. Using six socioeconomic indicators, Laaksonen *et al.* [8] identified a strong association between education, occupational status and current smoking. Cavelaars *et al.* [9] found higher rates of current and ever-smoking among less educated individuals in northern European countries. Barbeau *et al.* [10] found the same type of association in the United States, where they noted an increased prevalence of current smoking and an independent association between current smoking and lower-paid jobs, low education levels and lower income levels. Moreover, they found a positive association between success in quitting and socioeconomic resources. This last finding is supported by the studies of Borland *et al.* [11], and more recently Lee and Kahende [12], in which the authors found an association between certain socioeconomic indicators and the probability of successfully quitting.

In a recent review, Schaap and Kunst [13] noticed that the majority of studies on socioeconomic inequalities in smoking focused on education and used smoking prevalence as the outcome of interest. The authors emphasized the importance of analysing smoking inequalities with respect to other socioeconomic indicators and various smoking outcomes related to initiation and cessation. With this in mind, we decided to analyse the association between two socioeconomic indicators—education and income—and two outcomes related to smoking cessation: the odds of successfully quitting and the duration of abstinence. In a first step, we conducted multivariate logistic regressions to assess the socioeconomic differences between successful and unsuccessful quitters. Then, relying on detailed information about past smoking behaviour, we retrieved the abstinence episodes of both successful and unsuccessful quitters. The resulting information on time before relapse was analysed in the duration analysis framework. To our knowledge, ours is the first study to assess the simultaneous impact of several socioeconomic indicators on abstinence duration. This approach allowed us to conduct a comprehensive analysis of smoking cessation, relying on a more detailed temporal dimension. For both parts of the study we used pooled data from the 2001–2007 editions of the Swiss Tobacco Survey [14]. In each case, we controlled for potential confounders such as age, region, and other health-related behaviours. We investigated the following research questions: (1) Which aspects of social position are the strongest predictors of successful cessation? (2) Which socioeconomic factors influence the time before relapse? (3) Do the socioeconomic determinants of successful cessation and abstinence duration differ between men and women?

## 2. Method

### 2.1. Data

We pooled data from the 2001–2007 editions of the Swiss Tobacco Monitoring Survey [14], a nationwide, cross-sectional survey of 14–65 year-olds conducted annually in Switzerland since 2001. Each quarter about 2,500 individuals are interviewed by phone in French, German or Italian, resulting in a total of about 10,000 observations per year (several subgroups of the population were oversampled—men aged 14–24, women aged 14–44 and individuals from the Italian and French linguistic regions). Combined, the seven cross-sections consisted of 70,216 respondents. In addition to demographic and socioeconomic information, the database contains a large number of variables related to smoking history and current smoking behaviour. From the base sample, we constructed one subsample which consisted of current and former smokers, aged 18 and over, who had recently attempted to quit.

### 2.2. Variables

#### 2.2.1. Outcome Variables

To distinguish between successful and unsuccessful quitters we had to exploit information about individual smoking history. We based the construction of this variable on the work of Lee and Kahende [12], who conducted a similar type of analysis in the United States. Unsuccessful quitters were defined as current smokers who had tried to quit at least once during the last 12 months, *i.e.*, current smokers who answered yes to the question “Did you seriously try to quit smoking during the past 12 months?” Successful quitters were defined as ex-smokers who quit between seven and sixty months ago (our analysis focuses on recent cessation activity, which is the reason why we did not include individuals who quit more than five years ago). As suggested by Lee and Kahende [12], we excluded smokers who quit in the past six months because the risk of relapse is often very high for these people. The dependant variable in the relapse analysis was the duration in days of the longest quit attempt (also referred to as the duration of abstinence). Successful quitters were treated as censored observations since no relapse has been observed for them.

#### 2.2.2. Independent Variables

Demographic characteristics included age, gender and marital status. Age was categorised into three groups: 18–24, 25–44 and 45–65. Marital status was used to distinguish between married and non-married respondents. Socioeconomic variables included education level and household income. Education was divided into three categories as follows: basic education (no education or compulsory schooling only), secondary education (apprenticeship, vocational school and secondary school) and higher education (advanced professional training, college and university). Net monthly household income categories were: Swiss francs (CHF) 0–4,000, CHF 4,001–8,000 and CHF 8,000 and over. Many studies have shown a significant link between smoking and other health-related behaviours [15–18]. We chose to include heavy drinking and concern for healthy eating as independent variables in the models. These categories were defined by those who drank two or more alcoholic beverages per day and by a positive response to the question “Do you try to eat a healthy diet?” We also included a dummy variable that was equal to one if the individual lived in a region where tobacco control was intensive at the time of the study [there are seven regions in Switzerland (Lake Geneva Region, Mittelland, North West Switzerland, Zurich, Eastern Switzerland, Central Switzerland and Ticino) some of them have implemented more stringent tobacco control policies]. Finally we included a dummy variable for each interview year to account for potential trends.

### 2.3. Statistical Analysis

We first conducted a simple descriptive analysis to assess the association between socioeconomic indicators and the variable of interest in each analysis (successful cessation and abstinence duration). The differences between each socioeconomic subgroup were computed in both absolute and relative terms. To evaluate the simultaneous impact of socioeconomic characteristics on successful cessation while controlling for potential confounders, we conducted multivariate logistic regressions for men and women separately. Analysis of relapse was performed using the duration analysis framework, in which the variable of interest is a time period. In our case, the dependant variable was the duration, in days, of the longest quit attempt. Observations for which no relapse was observed are said to be censored and observations associated with unsuccessful quitters are uncensored (or complete). Among the numerous models available to analyse duration and its determinants, we opted for models in which the covariates are assumed to multiply the predicted time (accelerated failure-time models or AFT-AFT models are linear models of the logarithm of the survival time), in contrast with proportional hazard models in which the covariates are assumed to multiply the chance that an event occurs. Among the suitable distributions for AFT models, the log-logistic distribution is the most commonly used. The Weibull, exponential, log-normal, gamma or inverse Gaussian distributions are also appropriate. We estimated a series of AFT models by relying on various distributional assumptions. Since the different distributional assumptions have led to very similar estimates, we only report the results obtained with the log-logistic distribution. These models were applied to men and women separately. Our specifications accounted for unobserved heterogeneity (the unobserved heterogeneity was assumed to be Gamma distributed), and all estimations were performed using Stata version 10.0 (Stata Corp., Texas, USA).

## 3. Results

### 3.1. Study Population

In the 2001–2007 editions of the tobacco monitoring survey, 70,216 individuals completed the survey, among whom 63,520 were aged 18 and over. These individuals included 19,622 smokers (30.9%), 12,874 former smokers (20.3%) and 31,024 individuals who had never smoked (48.8%). The latter were excluded from the analysis, resulting in a base sample of 32,496 current or former smokers aged 18 and over. The successful cessation analysis compared the characteristics of former smokers who reported quitting seven to sixty months before the survey (N = 3,530) and those of unsuccessful quitters, defined as current smokers who had attempted to quit during the last twelve months (N = 4,145). For each analysis, our sample was limited to respondents who had no missing values for the relevant covariates, leaving 6,290 individuals for the cessation analysis (1,385 missing) and 6,136 individuals for the relapse analysis (1,539 missing). The majority of missing values were due to a lack of information about income.

### 3.2. Descriptive Analysis

#### 3.2.1. Successful Cessation Analysis

Both socioeconomic indicators were strongly associated with successful cessation (Table 1). The proportion of successful quitters was much higher among highly educated individuals than among respondents who had only completed compulsory education. We observed similar differences between the two extreme income groups.

#### 3.2.2. Abstinence Duration (Relapse Analysis)

**Figure 1:**
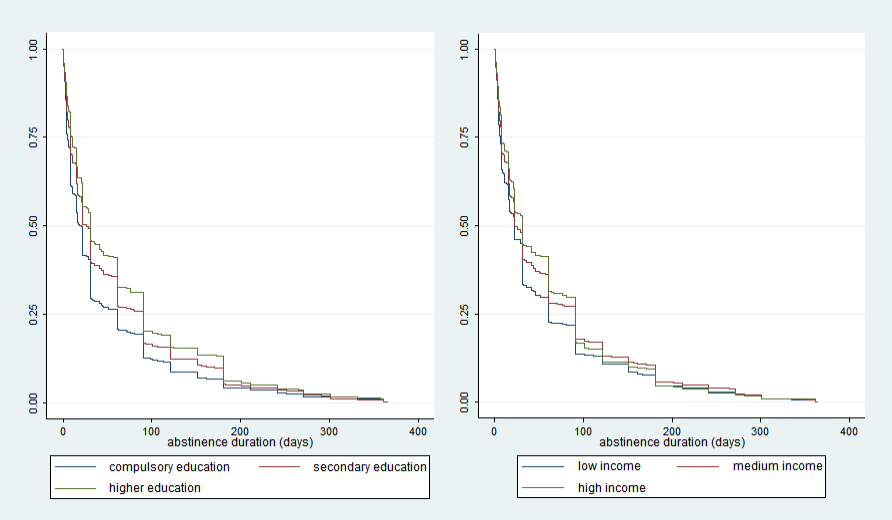
Non-parametric survival functions by education and income.

In Table 1, we observe that, among unsuccessful quitters, the mean time before relapse was 60% higher for highly educated individuals in comparison with individuals who had only completed compulsory education. The duration was 25% higher for the highest income group in comparison with the lowest income group. Figure 1 shows the non-parametric estimates of the survival functions [19] with respect to both socioeconomic indicators. The data indicate the proportion of the population of interest that had not relapsed at each observation time. A sharp decrease of the function at a specific time indicates that a large number of individuals relapsed at that time. We clearly see that the survival function for individuals with only a compulsory education is lower than the function associated with more highly educated individuals, indicating a higher relapse rate among less educated individuals. The same relationship was observed between high and low income individuals.

### 3.3. Multivariate Analysis

#### 3.3.1. Successful Cessation Analysis

Estimation results are shown in Table 2. We observed an important socioeconomic gradient in successful cessation with respect to both education level and income. In the group with higher education, the odds of being a successful quitter in comparison with the reference category (compulsory education) reached 1.39 for men and 1.78 for women. The influence of higher income levels is comparable in size for both subgroups (OR_men_ = 1.65 and OR_women_ = 1.47). We observed that the odds of being a successful quitter were linked with marital status for both men and women (OR_men_ = 1.36 and OR_women_ = 1.36).

A strong age effect was observed among men, indicating that older smokers are more than twice as likely to quit successfully when compared with smokers in the 18–24 age group (OR = 2.59). Women who were interested in healthy eating were more likely to quit in the long run (OR = 1.50). Excessive alcohol consumption and being in a region where prevention is intensive did not seem to significantly influence successful cessation.

#### 3.3.2. Abstinence Duration (Relapse Analysis)

As suggested by Cleves *et al.* [20], we reported the exponentiated coefficients, also known as time ratios, because of their ease of interpretation (Table 3). Time ratios represent the factor by which the expected abstinence duration (or time before relapse) is multiplied as a result of a one unit increase in the corresponding covariate. A time ratio of 1.2 associated with a dummy variable means that the expected abstinence duration of individuals for whom the dummy equals one is 20% higher than that of individuals in the reference category. The models consistently showed that more educated individuals, especially women, had longer abstinence duration. The expected abstinence duration of individuals with higher education is twice larger than the one associated with their less educated counterparts. The income level seemed to have a smaller, although significant, impact on abstinence duration among men and women, but only for high income individuals. Marital status seemed to have no significant impact on the time before relapse. Individuals in the older age group seemed to relapse more quickly than their younger counterparts. In the regions characterised by high prevention intensity, the abstinence duration was 70% longer for men and 100% longer for women. Both health-related behaviours (*i.e.*, excessive alcohol use and healthy diets) had a significant impact on the abstinence duration among men, where a negative effect for alcohol abuse and a positive effect for healthy dietary habits were observed. For women, excessive alcohol use didn’t seem to influence abstinence duration.

## 4. Discussion

Although socioeconomic inequalities in smoking were extensively documented, a large part of the studies focused on the association between one particular socioeconomic dimension—mostly the education level—and smoking prevalence (Schaap and Kunst [13]). The aim of our study was to analyse the simultaneous impact of education and income on smoking cessation, using the 2001–2007 editions of the Swiss Tobacco Survey. The cessation behaviour was assessed with two outcomes, resulting in two independent analyses. We first analysed the probability of being a successful quitter by means of multivariate logistic regressions. Then, using retrospective information about smoking behaviour, we analysed the time before relapse in the duration analysis framework. We found evidence of a socioeconomic gradient in successful cessation and abstinence duration with respect to education level and income for both men and women. The gradient associated with education is more pronounced for women than for men, while income seems to have a comparable impact in both groups.

Several shortcomings of our study should be noted. First, we pooled seven cross-sectional data sets and controlled for aggregate changes over time using year dummy variables. Each year a new random sample was drawn from the population, and the distributions of the variables probably changed over time. This could lead to biased estimates. Second, a large proportion of respondents did not provide any information about their income, significantly reducing our sample size. Third, we relied on self-reported information to determine smoking-related outcome variables, which are consequently likely to exhibit misreporting and recall bias. Although several studies confirm the reliability of self-reported smoking status (see e.g., Caraballo *et al.* [21]), we must interpret our results with caution, as we don’t know if this is the case with self-reported data on smoking cessation. Another important point is that the database fails to provide retrospective information about variables such as consumption level (number of cigarettes smoked per day) or dependence (Fagerström test for nicotine dependence) for former smokers. We were therefore unable to control for these potentially influential factors. In addition, information about smoking cessation therapies was not exploited because it was available only for two observation years. Finally, as mentioned by Piasecki *et al.* [22] in their article about relapse: *“relapse to smoking is a dynamic process that may unfold idiosyncratically, and no single metric can perfectly summarise the relapse process.”* The authors mention the problem of renewed attempts that are not taken into account in duration analyses of relapse, as was the case in our study.

The policy implications of this study are important. We have shown that there is a socioeconomic gradient with respect to education and income in two outcomes associated with smoking cessation: the probability to quit successfully and the duration of abstinence. Lower success rates and shorter abstinence durations are apparent among lower socioeconomic groups in comparison with upper groups. The lag between smoking and its related diseases is important; and we may thus expect to observe an increasing prevalence of smoking-related disease among lower socioeconomic groups in the near future. Increasing cessation success rates and prolonging the duration of abstinence (with definitive abstinence as a final objective) in such disadvantaged groups could lead to an important reduction in smoking inequalities and a reduction of health inequalities in the longer term. Appropriate policies targeted at lower social classes have to be implemented. Available products aimed at improving cessation success can considerably enhance long-term abstinence rate [23,24]. However, they are expensive and are not reimbursed by the social health insurance programs available in Switzerland. To improve success rates and extend abstinence periods, smoking cessation therapies including nicotine replacement therapies, nicotine-free medications and counselling should be made more accessible to lower socioeconomic groups.

## Figures and Tables

**Table 1:** Descriptive analysis of outcomes across socioeconomic subgroups.

	**Cessation analysis (N = 6,290)**			**Abstinence duration analysis (N = 3,209)**		
	% of successful quitters	absolute diff. in the proportion of successful quitters compared to reference category (pp.)	relative diff. compared to reference category (%)	mean duration, in days, of longest quit attempt (unsuccessful quitters)	absolute diff. in mean duration compared to reference category	relative diff. compared to reference category (%)
**Education Level**						
Compulsory	34.5	-	-	61.8	-	-
Secondary	45.4	+10.9	+31.6	86.6	+24.8	+40.1
Higher	54.2	+19.7	+57.1	99.4	+37.6	+60.8
**Household Income**						
Up to 4,000	39.6	-	-	79.4	-	-
4,000–8,000	47.5	+7.9	+19.9	87.1	+7.7	+9.7
8,000 +	53.9	+14.3	+36.1	100.5	+21.1	+26.6

**Table 2:** Successful cessation—Multivariate logistic regressions.

	**Odds of successfully quitting**	
	*Men*	*Women*
**Education**		
Compulsory Secondary Higher	1.00 (ref.) 1.13 (0.92) 1.39* (2.15)	1.00 (ref.) 1.40** (3.10) 1.78*** (4.49)
**Household Income**		
Up to 4000	1.00 (ref.)	1.00 (ref.)
4–8 8+	1.13 (1.11) 1.65*** (3.98)	1.27** (2.76) 1.47*** (3.58)
**Age**		
18–24 25–44 45–65	1.00 (ref.) 2.20*** (5.83) 2.59*** (6.59)	1.00 (ref.) 1.32** (2.38) 1.18 (1.30)
**Marital status**		
Non-married Married	1.00 (ref.) 1.36* (3.46)	1.00 (ref.) 1.36*** (4.17)
**Heavy drinking (“regular drinker”)**		
No Yes	1.00 (ref.) 0.91 (−1.14)	1.00 (ref.) 1.00 (0.02)
**Interest in healthy diet**		
No Yes	1.00 (ref.) 1.08 (0.82)	1.00 (ref.) 1.50** (3.30)
**Region with high prevention intensity**		
No Yes	1.00 (ref.) 1.09 (1.09)	1.00 (ref.) 0.99 (−0.04)
*N*	2,691	3,599

Note: t-statistics in parentheses;

* p < 0.05,

** p < 0.01,

*** p < 0.001; Year dummies not reported.

**Table 3:** Abstinence duration models—Accelerated failure time (log-logistic distribution).

	***Time ratios***	
	*_Men*	*_Women*
**Education (ref: compulsory)**		
Secondary	1.28 (1.68)	1.61*** (3.69)
Higher	2.16*** (4.19)	2.27*** (4.89)
**Income (ref: low income)**		
Middle income High income	1.23 (1.58) 1.47* (2.47)	1.12 (0.96) 1.44* (2.47)
**Age (ref: 18–24)**		
25–44	0.93 (−0.50)	0.82 (−1.37)
45–65	0.57** (−3.34)	0.48*** (−4.84)
**Marital status (ref: non-married)**		
Married	0.99 (−0.05)	0.99 (−0.02)
**Heavy drinking (ref: no)**		
Yes	0.76* (−2.55)	0.83 (−1.49)
**Interest in healthy diet (ref: no)**		
Yes	1.47** (3.24)	1.35* (2.01)
**Region with high prevention intensity (ref: no)**		
Yes	1.73*** (5.26)	2.04*** (7.26)
*N*	2,614	3,522

Note: t-statistics in parentheses

* p < 0.05,

** p < 0.01,

*** p < 0.001; Constants, scale parameters and year dummies not reported.
